# Voluntary Modulation of Hemodynamic Responses in Swallowing Related Motor Areas: A Near-Infrared Spectroscopy-Based Neurofeedback Study

**DOI:** 10.1371/journal.pone.0143314

**Published:** 2015-11-17

**Authors:** Silvia Erika Kober, Bettina Gressenberger, Jürgen Kurzmann, Christa Neuper, Guilherme Wood

**Affiliations:** 1 Department of Psychology, University of Graz, Graz, Austria; 2 BioTechMed-Graz, Graz, Austria; 3 Laboratory of Brain-Computer Interfaces, Institute for Knowledge Discovery, Graz University of Technology, Graz, Austria; University of Toyama, JAPAN

## Abstract

In the present study, we show for the first time that motor imagery of swallowing, which is defined as the mental imagination of a specific motor act without overt movements by muscular activity, can be successfully used as mental strategy in a neurofeedback training paradigm. Furthermore, we demonstrate its effects on cortical correlates of swallowing function. Therefore, *N* = 20 healthy young adults were trained to voluntarily increase their hemodynamic response in swallowing related brain areas as assessed with near-infrared spectroscopy (NIRS). During seven training sessions, participants received either feedback of concentration changes in oxygenated hemoglobin (oxy-Hb group, *N* = 10) or deoxygenated hemoglobin (deoxy-Hb group, *N* = 10) over the inferior frontal gyrus (IFG) during motor imagery of swallowing. Before and after the training, we assessed cortical activation patterns during motor execution and imagery of swallowing. The deoxy-Hb group was able to voluntarily increase deoxy-Hb over the IFG during imagery of swallowing. Furthermore, swallowing related cortical activation patterns were more pronounced during motor execution and imagery after the training compared to the pre-test, indicating cortical reorganization due to neurofeedback training. The oxy-Hb group could neither control oxy-Hb during neurofeedback training nor showed any cortical changes. Hence, successful modulation of deoxy-Hb over swallowing related brain areas led to cortical reorganization and might be useful for future treatments of swallowing dysfunction.

## Introduction

Prior neurofeedback (NF) studies could show that participants can learn enhanced voluntary control over task-specific activation in the somatomotor cortex by motor imagery of hand or foot movements [1–7]. Motor imagery, which is defined as the mental imagination of a specific motor act without overt movements by muscular activity, generally activates similar brain areas as motor execution [8–12]. Getting real-time feedback about the level of activation in the somatomotor cortex can lead to motor improvements, which are also associated with changes in corresponding brain activation patterns [1,3,8,13–18]. Hence, voluntary modulation of brain signals by means of NF training using mental imagery of a specific motor task leads to neuronal reorganization. Based on these findings, real-time feedback applications using mental practice with motor imagery turned out to be valuable tools in motor rehabilitation of upper and lower limb functions [8,19–21]. However, to date motor imagery based NF training paradigms are restricted to the imagination of hand and foot movements [1–9,17,18,22]. To the best of our knowledge, motor imagery of swallowing movements has not been used as mental strategy in NF training studies before. There is evidence that motor execution (ME) and motor imagery (MI) of swallowing movements activate comparable brain areas as well. Kober et al. (2014, 2015) could show that ME and MI of swallowing led to hemodynamic signal changes over the inferior frontal gyrus (around BA 44, bilaterally) in healthy participants as well as in stroke patients with swallowing difficulties [12,23]. In the present study, we investigated for the first time whether participants are also able to voluntarily modulate their hemodynamic response in swallowing related brain areas when getting real-time feedback. Therefore, we used a near-infrared spectroscopy (NIRS)-based NF training paradigm in which participants imagined swallowing movements. Our aim was to evaluate the effects of NIRS-based NF training on cortical activation patterns underlying swallowing function.

In the present study, we decided to use NIRS-based NF training. The majority of NF training studies are based on the electroencephalogram (EEG), but EEG parameters are not the only physiological parameters that reflect functioning of the brain. Hemodynamic or metabolic activity of the brain measured by functional magnetic resonance imaging (fMRI) or NIRS can be effectively used as NF signal as well [1,7]. NIRS is a relatively new, non-invasive optical neuroimaging technique that measures concentration changes of oxygenated hemoglobin (oxy-Hb) and deoxygenated hemoglobin (deoxy-Hb) in the cerebral vessels based on their different absorption spectra for light in the near-infrared range [24]. The typical hemodynamic response to mental activation is the measurement basis of NIRS, such as for fMRI [25]. The hemodynamic parameters can be fed back to the participants and, consequently, can be used for voluntary control of brain functions [1,3,4,7,26]. Healthy participants as well as patients with brain lesions can learn enhanced voluntary control over the NIRS signal [1,3,13–16,18]. Note that these prior NIRS-based NF training studies only used the oxy-Hb signal as feedback signal. Probably only the oxy-Hb level was chosen for feedback as this was noted to exhibit larger signal changes than deoxy-Hb [27]. Furthermore, there is evidence that the oxy-Hb signal is the most sensitive indicator of changes in cerebral blood flow (CBF). The CBF is an indicator of cortical activation. In contrast, the direction of changes in deoxy-Hb is determined by the degree of changes in venous blood oxygenation and volume [28]. The link between oxy-Hb and CBF might also be a reason why oxy-Hb has often been used as feedback signal in NIRS-based NF training studies. Since motor imagery of swallowing has never been used as mental strategy in NF training studies before, in the present proof-of-principle study we used both, the oxy- and deoxy-Hb as feedback signals. On the one hand, we investigated the specific trainability of oxy- and deoxy-Hb. We wanted to know whether participants can learn to voluntarily modulate both signals by means of NF training. On the other hand, we evaluated their specific effects on brain activation patterns underlying the swallowing process.

Generally, swallowing is a complex and fundamental neuromuscular activity consisting of voluntary (oral phase) and involuntary (pharyngeal and esophageal phase) phases leading to activation patterns in multiple brain regions [29–31]. Therefore, a number of neurological disorders interfere with the swallowing process and cause dysphagia, having negative impact on the quality of life and health of affected people [32,33]. Using NIRS, only swallowing related brain activation patterns in cerebral cortical regions can be assessed. NIRS measures changes in hemodynamic responses only a few centimeters (0.5–2 cm) from the surface of the head [34]. Hence, swallowing centers in deeper brain regions such as the brainstem, which is mainly associated with reflexive components of swallowing (involuntary, pharyngeal and esophageal phase), cannot be examined in the present study [32]. However, reflexive and volitional swallowing is also represented in cerebral cortical regions [30]. Cerebral cortical representations of active swallowing in healthy participants have been frequently investigated using neuroimaging techniques such as functional magnetic resonance imaging (fMRI), positron emission tomography (PET), transcranial magnetic stimulation (TMS), magnetoencephalography (MEG), and electroencephalography (EEG) [13,30–32,35–51]. There are also a few prior NIRS studies investigating cortical correlates of swallowing [12,23,52,53]. These prior neuroimaging studies provide evidence that reflexive swallowing leads to bilateral activity concentrated to the primary sensory/motor regions, while volitional swallowing leads to bilateral activation foci over the insular cortex, the frontal operculum, prefrontal, cingulate, and parieto-occipital regions in addition to the primary sensory/motor cortex [30,31]. Kern et al. (2001) hypothesized that non-sensory/motor regions activated during active volitional swallowing may represent swallowing related intent, planning, decision making, memory, as well as information processing related to deglutition [30]. Hence, motor imagery of swallowing might also involve such top-down control mechanisms involved in the voluntary/oral swallowing phase.

Summing up, in the present investigation we implemented a NIRS-based real-time feedback system using mental imagery of swallowing movements. First, we addressed the question whether both hemodynamic parameters, oxy- and deoxy-Hb, can be modulated voluntarily by means of real-time feedback, when participants imagine swallowing. Previous NIRS-based NF studies successfully used oxy-Hb as feedback signal during motor imagery of limb movements [1–4]. Prior fMRI-based NF training studies demonstrated that participants can learn to control the BOLD signal, which corresponds to the deoxy-Hb signal [7,54–56]. Hence, based on these prior NIRS and fMRI NF studies, we hypothesized that participants can learn to voluntary modulate both NIRS parameters. Second, we focused on the effects of NIRS-based NF training on swallowing related brain activation patterns. Therefore, we measured the cortical correlates of ME and MI of swallowing before and after NF training. In accordance to the findings of prior NF studies using mental imagery of limb movements, we expect that NF training using mental imagery of swallowing movements might lead to cortical reorganization in swallowing related brain areas [1–4,13,14,18,23].

## Materials and Methods

### 2.1 Participants

Twenty healthy adults (10 female, 10 male) took part in this study. All participants gave written informed consent, had no history of neurological, psychiatric, respiratory, or swallowing disorders and had normal or corrected-to-normal vision. The study was approved by the Ethics Committee of the University of Graz, Austria (reference number GZ. 39/25/63 ex 2013/14) and is in accordance with the ethical standards of the Declaration of Helsinki. Participants were randomly assigned to one of two neurofeedback (NF) training groups, although the two groups were matched for gender: an oxy-Hb group (5 females, 5 males, mean age = 23.8 yrs., *SE* = 0.47) and a deoxy-Hb group (5 females, 5 males, mean age = 25.7 yrs., *SE* = 1.20). The oxy-Hb group got real feedback about relative concentration changes in their oxy-Hb level, whereas the deoxy-Hb group got real feedback about changes in deoxy-Hb over the inferior frontal gyrus bilaterally during movement imagery. Participants did not know which group they were in, nor did they know about the oxy-versus-deoxy-Hb study design. The two feedback groups did not differ in their motor imagery ability as assessed with the MIQ-R [57]: oxy-Hb group–visual imagery: *M* = 23.70, *SE* = 1.03; oxy-Hb group–kinesthetic imagery: *M* = 24.70, *SE* = 0.96; deoxy-Hb group–visual imagery: *M* = 23.00, *SE* = 0.91; deoxy-Hb group–kinesthetic imagery: *M* = 25.00, *SE* = 0.80 (visual imagery: *t*(18) = -0.51, *ns*.; kinesthetic imagery: *t*(18) = 0.24, *ns*.). Furthermore, groups did not differ in their swallowing performance as assessed with a 100-ml water swallowing test [58]: oxy-Hb group: *M* = 17.81 ml/s, *SE* = 1.12; deoxy-Hb group: *M* = 18.91 ml/s, *SE* = 1.56 (*t*(18) = 0.57, *ns*.). For the 100-ml water swallowing test, participants were seated upright and were ask to place a glass of 100 ml of still and room-temperature water to their lips. After receiving a go-signal, they drank the water as quickly as possible. A stop-watch was used to measure the swallowing time, measured from the go-signal to the end of the last swallow. Swallowing speed is defined as the amount of drunken water divided by the elapsed time (ml/s). All participants of the present study showed a normal swallowing speed >10 ml/s [58].

### 2.2 NIRS-based neurofeedback training

#### 2.2.1 Neurofeedback training sessions

**Fig 1:**
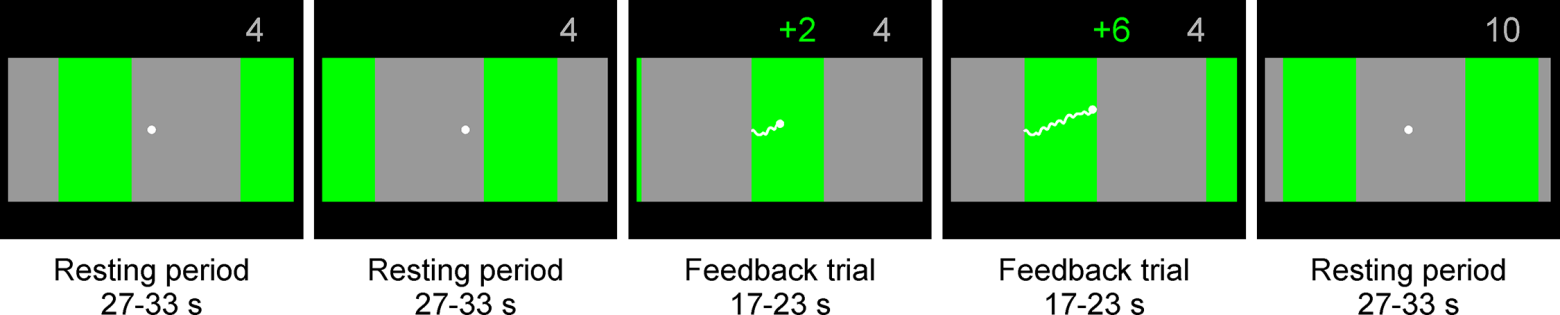
Feedback screen. On the background of the feedback screen, either green or gray stripes moved from the right to the left side of the screen with a constant speed. During the resting period, the white dot in the middle of the screen overlapped a gray stripe of the moving background and was vertically fixed in the center of the screen (first, second and fifth example screens in the figure). During the feedback trials, the white dot overlapped a green stripe of the moving background and participants tried to move the white dot up by motor imagery of swallowing (third and fourth example screens in the figure). When they were successful, the number of reward points increased (green reward points indicated number of reward points obtained in the actual feedback trial, gray reward points indicated sum of all obtained reward points of all previous feedback trials).

**Fig 2:**
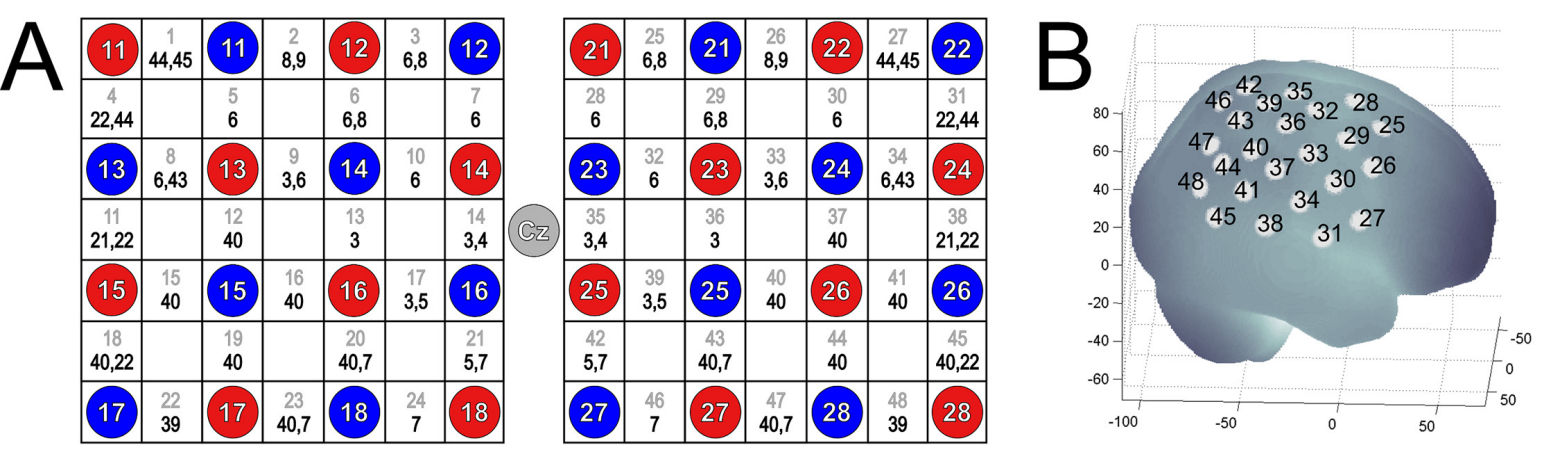
Position of the optode probe set on the head. (A) Channel configuration of the optode probe set (2x4x4). The gray numbers in the white rectangles represent the NIRS channel number and the corresponding Brodmann areas are shown in black. The NIRS probe set of 48 channels was positioned over right and left motor cortex. Red and blue circles illustrate positions of NIRS sensors and detectors, respectively. According to the international 10–20 placement system we used Cz, T7 and T8 as marker positions for ensuring replicable placements of the optodes. (B) Projections of the 48 NIRS channel positions (white points) on the cortical surface over the right hemisphere. NIRS positions are overlaid on a MNI-152 compatible canonical brain that is optimized for NIRS analysis according to a procedure of Singh et al. (2005) [63].

Seven neurofeedback (NF) training sessions were carried out three to five times a week, each session on a different day. Each training session took approximately 20 minutes and included 25 feedback trials. Participants got visual feedback about their relative concentration changes in oxy- or deoxy-Hb, respectively. This relative NIRS concentration changes were reflected by a vertically moving white dot in the middle of the feedback screen. On the background, either green or gray stripes moved from the right to the left side of the screen with a constant speed. Whenever the white dot overlapped with a gray stripe of the moving background, participants were instructed to relax. This resting period had a variable duration of 27 to 33 seconds. The white dot was also vertically fixed in the center of the screen during this resting condition. Whenever the white dot overlapped with a green stripe of the moving background, participants were instructed to imagine swallowing. During this feedback trial, the white dot moved up and down, depending on the level of oxy- or deoxy-Hb in swallowing related motor areas. The duration of each feedback trial varied between 17 and 23 seconds. The feedback screen during the resting periods and the feedback trials is illustrated in Fig 1. In the oxy-Hb group, difference values between oxy-Hb over the bilateral inferior frontal gyrus (average signal of optode number 1, 4, 27, 31) and oxy-Hb over more posterior sites (average signal of optode number 18, 22, 45, 48) corresponded to the vertical position of the white point on the screen. In contrast, in the deoxy-Hb group, difference values between deoxy-Hb over the bilateral inferior frontal gyrus (average signal of optode number 1, 4, 27, 31) and deoxy-Hb over more posterior placed optodes (average signal of optode number 18, 22, 45, 48) corresponded to the vertical position of the white point on the screen (see Fig 2). The feedback optodes were chosen in accordance to findings of a previous NIRS study, in which the bilateral inferior frontal gyrus (optode number 1, 4, 27, 31) was most active during MI and ME of swallowing, whereas oxy- and deoxy-Hb over more posterior sites (e.g. optode number 18, 22, 45, 48) did not show any significant signal changes during ME and MI of swallowing [12]. If there was a stronger increase in oxy-/deoxy-Hb over the inferior frontal gyrus (optode number 1, 4, 27, 31) compared to more posterior sites (optode number 18, 22, 45, 48) during motor imagery, the white point moved up, otherwise it moved down. The course of the feedback was indicated by a white trace that was drawn by the dot during the imagery condition. The participants’ task was to move the white dot up. They were rewarded with a numerical score if they moved the dot up successfully.

#### 2.2.2 NIRS neurofeedback model

The NIRS NF model was realized with Simulink (MATLAB–The MathWorks) and is described in more detail in a previous study [1]. For processing the NIRS signal online, HITACHI Medical Co. (Japan) provided a real-time output solution for the ETG-4000. As a first step, the NIRS raw signal was filtered online with a 0.01 Hz high pass and a 1.5 Hz low pass filters. Based on a previous study [12], predefined channels of the bilateral inferior frontal gyrus (optode number 1, 4, 27, 31) and more posterior sites (optode number 18, 22, 45, 48) were smoothed using 2 s moving average window. Finally, the difference in oxy-/deoxy-Hb between the averaged feedback channels was calculated. Calculating difference values in oxy-/deoxy-Hb between the inferior frontal gyrus and more posterior sites reduces the influence of physiological artifacts such as respiration or pulse and extra brain artifacts such as skin blood flow that might confound signal changes in oxy-/deoxy-Hb [59,60]. Changes in these difference values in oxy-/deoxy-Hb during the movement imagery period were shown on a screen as a visual feedback (Fig 1).

### 2.3 Pre-post assessment: Brain activation patterns during motor execution (ME) and motor imagery (MI) of swallowing

Before and after seven sessions of NF training (pre- and post-assessment), participants performed an active water swallowing task and a motor imagery task to assess the cortical activation patterns during motor execution (ME) and motor imagery (MI) of swallowing using NIRS. Therefore, participants were instructed to voluntarily swallow small gulps of room-temperature water that was drawn through a 3-mm-diameter flexible tube attached to a 1 liter bottle of water during the ME task. During one 15 s ME trial, participants took about 5 to 6 sips of water. During the MI task, participants should imagine to swallow but not execute any swallowing movement. Participants were instructed to imagine to take 5 to 6 sips through the straw and to swallow each sip like in the ME task. Each MI trial lasted 15 seconds. In sum, 20 MI and 20 ME trials were presented in a randomized order. Between the MI and ME trials, a fixation cross appeared at the screen with a variable duration of 28 to 32 seconds. During these resting trials, participants were instructed to relax and to avoid swallowing as much as they could. Participants were shortly trained in both tasks at the beginning of the experimental session to accustom themselves to the timing of the trials before starting the MI/ME tasks.

### 2.4 NIRS-recordings and -analysis

To assess relative concentration changes of oxygenated (oxy-Hb) and deoxygenated hemoglobin (deoxy-Hb) over swallowing related areas of the brain during the NF training as well as during the MI/ME task during the pre- and post-assessment, NIRS measurements were performed on a continuous wave system (ETG-4000, Hitachi Medical Co., Japan) using two 4×4 optode probe sets (consisting of 16 photo-detectors and 16 light emitters) resulting in a total of 48 channels (see Fig 2). The ETG-4000 uses two different wavelengths (695±20 nm and 830±20 nm). To prevent crosstalk, its frequency is modulated for wavelengths and channels [9,61]. Since continuous wave systems, such as the ETG-4000, cannot measure the optical path length [62], the scale unit is molar concentration multiplied by the unknown path length (mM/mm) [9]. The distance between the mounted optodes was 3 cm. The sampling rate of the NIRS system was set to 10 Hz. The channel configuration of the NIRS probe set is illustrated in Fig 2. Based on the findings of a previous study [12], in which we investigated the hemodynamic changes in the brain in response to ME and MI of swallowing using NIRS, the probe set was positioned over the motor cortex area. In accordance to the international 10–20 placement system, we used Cz, T7 and T8 as marker positions to place the probe set. The NIRS channels that were found to be most active during motor imagery and motor execution of swallowing (1, 4, 27, 31) around the bilateral inferior frontal gyrus [12] were used as NIRS feedback channels.

The MNI coordinates of the NIRS channels were assessed using ELPOS (zebris Medical GmbH), a system to determine 3D coordinates of EEG/NIRS electrode/optode positions with high accuracy based on the run-time measurement of ultrasonic pulses. Afterwards, a coordinate-based system was used to retrieve brain labels from the 1988 Talairach Atlas, called the Talairach Daemon [64,65]. In Fig 2A, the anatomic labels (Brodmann areas, Talairach Daemon) are indicated for each NIRS channel, averaged across all participants. Note that for some NIRS channels more than one Brodmann area (BA) is determined. This is a result of the differences in the participants’ sizes and shapes of the head.

For offline analysis of the NIRS signal, we focused on changes in oxy-Hb and deoxy-Hb over the bilateral inferior frontal gyrus (left hemisphere average channel 1 and 4; right hemisphere average channel 27 and 31). Before statistical analysis, the NIRS raw signal was preprocessed. Specifically, the raw data were corrected from artifacts (criterion for rejection: amplitude of Hb-signal > +/- 3 SD; visual inspection) and filtered with a 0.01 Hz high pass filter to remove baseline drifts and a 0.90 Hz low pass filter. To control for active movements during the MI task and also during the NF training sessions, the activity of the suprahyoid muscles (including mylohyoid, genohyoid, and anterior digastric muscles), which are involved during swallowing, was recorded using the electromyogram (EMG) [66]. Generally, EMG activity that is assessed at the suprahyoid muscles provides considerable information about the oropharyngeal swallowing phase [66]. EMG electrodes were placed at the right digastric muscle (anterior belly) and EMG raw signals were sampled at 256 Hz using a g.tec amplifier [66,67]. The recorded EMG signal was used to identify movement artifacts in the NIRS signal during artefact rejection. All trials with movement artefacts were removed from further analysis. For each task, the time courses of oxy-Hb and deoxy-Hb were averaged. Task-related concentration changes of oxy-Hb and deoxy-Hb were referred to a 5-s baseline interval prior to the task (seconds -5 to 0). For statistical analysis, oxy-Hb and deoxy-Hb during the task condition were averaged, respectively (seconds 5 to 20 after motor imagery onset for the NF training sessions, and for the pre-post ME/MI task seconds 5 to 15 after task onset for the active task period, and seconds 15 to 25 after task onset for the subsequent resting period). Note that for the ME/MI task, we analyzed NIRS signal changes during the active task period (seconds 5 to 15 after task onset) as well as NIRS signal changes directly after the active task period (seconds 15 to 25 after task onset) since prior studies indicated a prolonged NIRS time course for swallowing tasks that exceeds the active task period [12,23,52,53].

### 2.5 Statistical analysis

In order to analyze the NF training performance, the time course of oxy- and deoxy-Hb values over the seven NF training sessions was analyzed. Therefore, we conducted regression analyses separately for each group (predictor variable = session number; dependent variable = average of oxy-Hb or deoxy-Hb for the time interval 5–20 s after motor imagery onset during NF training).

To identify the NIRS channels that showed the strongest NIRS signal change (oxy- and deoxy-Hb) during ME/MI of swallowing, we used the False Discovery Rate (FDR) method to control the proportion of false positives among the channels that are detected as significant [68]. For this analysis, we averaged the NIRS signal (either oxy- or deoxy-Hb) for the time period 5–25 seconds after task onset separately for each of the 48 NIRS channels and the ME/MI task. Based on the results of the FDR analysis, channels that showed the strongest signal change during ME/MI were merged to two regions of interest (ROI) for further analysis: left inferior frontal region (LF), average channel 1, 4; right inferior frontal region (RF), average channel 27, 31.

To analyze differences in brain activation patterns during ME and MI of swallowing over the inferior frontal gyrus between the pre- and post-assessment, 2x2x2x2 analysis of variance (ANOVA) for repeated measures was applied including the within-subject factors PRE-POST (pre- vs. post-assessment), TASK (motor imagery vs. execution), HEMI (LF vs. RF), and TIME (active task = average seconds 5–15 after task onset vs. subsequent resting period = average seconds 15–25 after task onset) separately for oxy- and deoxy-Hb values and for both groups. For the ANOVA models the probability of a Type I error was maintained at 0.05. For posthoc analysis, Bonferroni corrections for multiple comparisons were applied.

## Results

### 3.1 NIRS NF training performance

To assess the NF training performance, the time course of oxy- and deoxy-Hb values over the seven NF training sessions was analyzed. The results of the regression analysis revealed that the deoxy-Hb group was successful in increasing the level of deoxy-Hb over the bilateral inferior frontal gyrus by means of NIRS-based NF training. For the deoxy-Hb group, the regression model for the dependent variable deoxy-Hb was significant (*F*(1,5) = 30.04, *p* < 0.01). With this regression model, 86% of variance of deoxy-Hb values over the seven training sessions could be explained. The regression model for the dependent variable oxy-Hb was significant for the deoxy-Hb group, too (*F*(1,5) = 47.63, *p* < 0.01). While deoxy-Hb increased over the seven NF training sessions, oxy-Hb decreased significantly in this group. Ninety-one percent of variance of oxy-Hb values over the seven training sessions could be explained by the regression model. In contrast, the oxy-Hb group was not able to linearly increase oxy-Hb over the NF training course. The regression model for the dependent variable oxy-Hb was not significant in the oxy-Hb group (*F*(1,5) = 0.02, *ns*.). However, the oxy-Hb group showed a linear increase in deoxy-Hb values (*F*(1,5) = 11.41, *p* < 0.05). With this regression model, 70% of variance of deoxy-Hb values over the seven training sessions could be explained.

**Fig 3:**
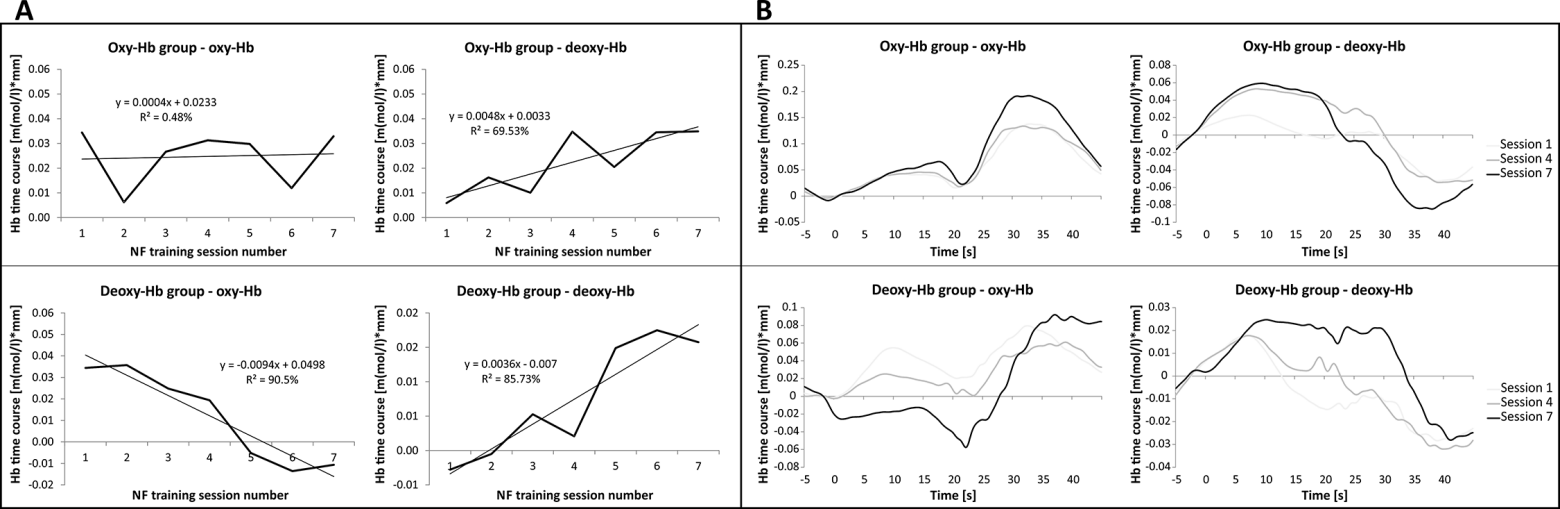
NIRS NF training performance. A) NF training performance (relative concentration changes in oxy- and deoxy-Hb over the bilateral inferior frontal gyrus during the NF training task, average second 5 to 20 after task onset) over the seven NF training sessions and the results of the regression analysis, presented separately for the oxy-Hb group (upper panel) and deoxy-Hb group (lower panel). B) NIRS time course during the first, fourth and last NF training session averaged for NIRS channels over the bilateral inferior frontal gyrus, presented separately for both groups. Note that the NF training task started at second 0 and ended after 17–23 seconds.

In Fig 3A, the NF training performance over the seven NF training sessions and the results of the regression analysis can be found, presented separately for each group and each NIRS parameter. Fig 3B illustrates the NIRS time course during the first, fourth and last NF training session averaged for NIRS channels over the bilateral inferior frontal gyrus, separately for both groups.

### 3.2 Pre-post comparison: Brain activation patterns during motor execution (ME) and motor imagery (MI) of swallowing

**Fig 4:**
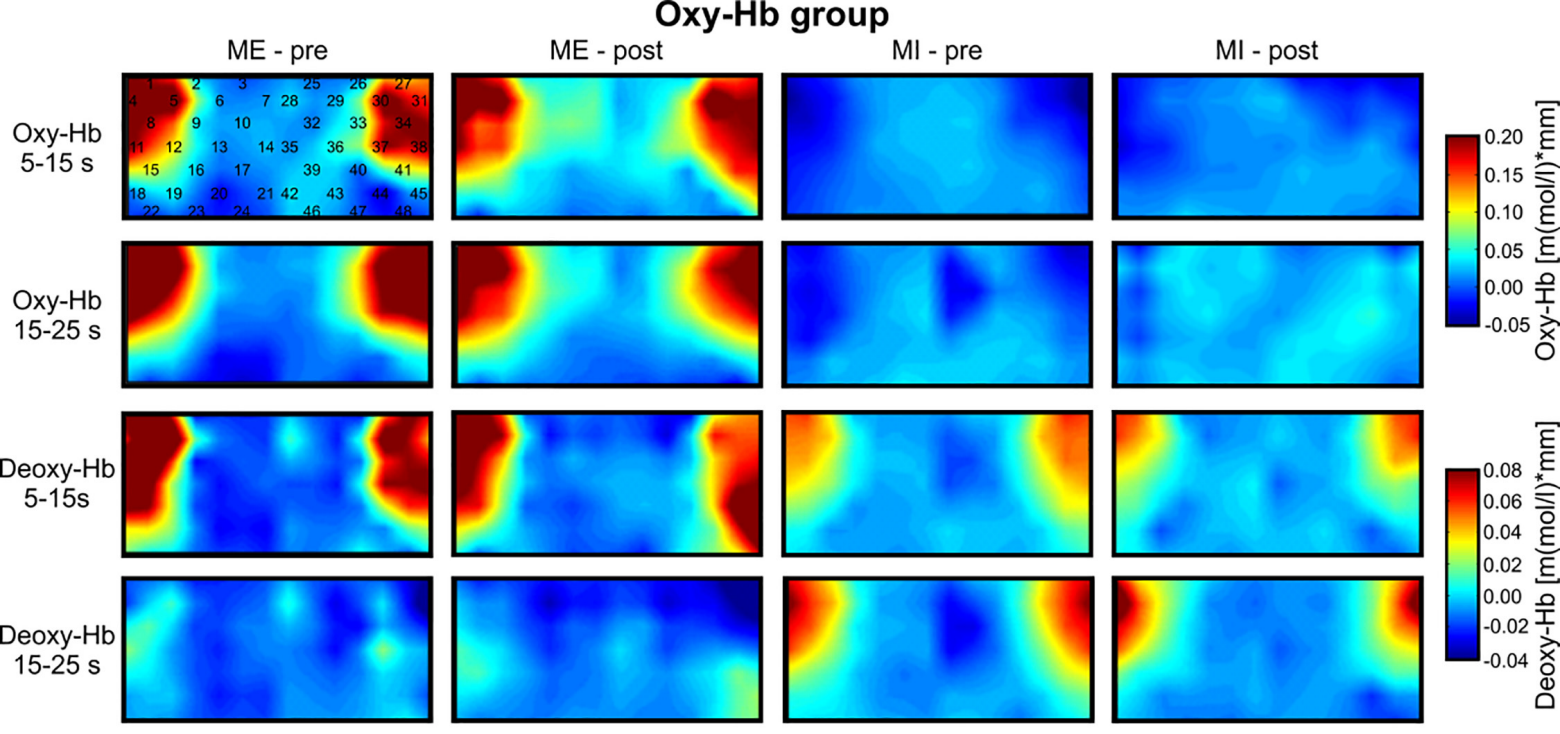
Topographical maps of oxy-Hb. Grand average topographical maps of oxy- and deoxy-Hb during motor execution (ME) and motor imagery (MI) for the oxy-Hb group, presented separately for the task interval (second 5–15 after task onset) and the pause interval (second 15–25 after task onset) and for the pre- and post-test. In the upper left map, the 48 NIRS channel locations are additionally marked.

**Fig 5:**
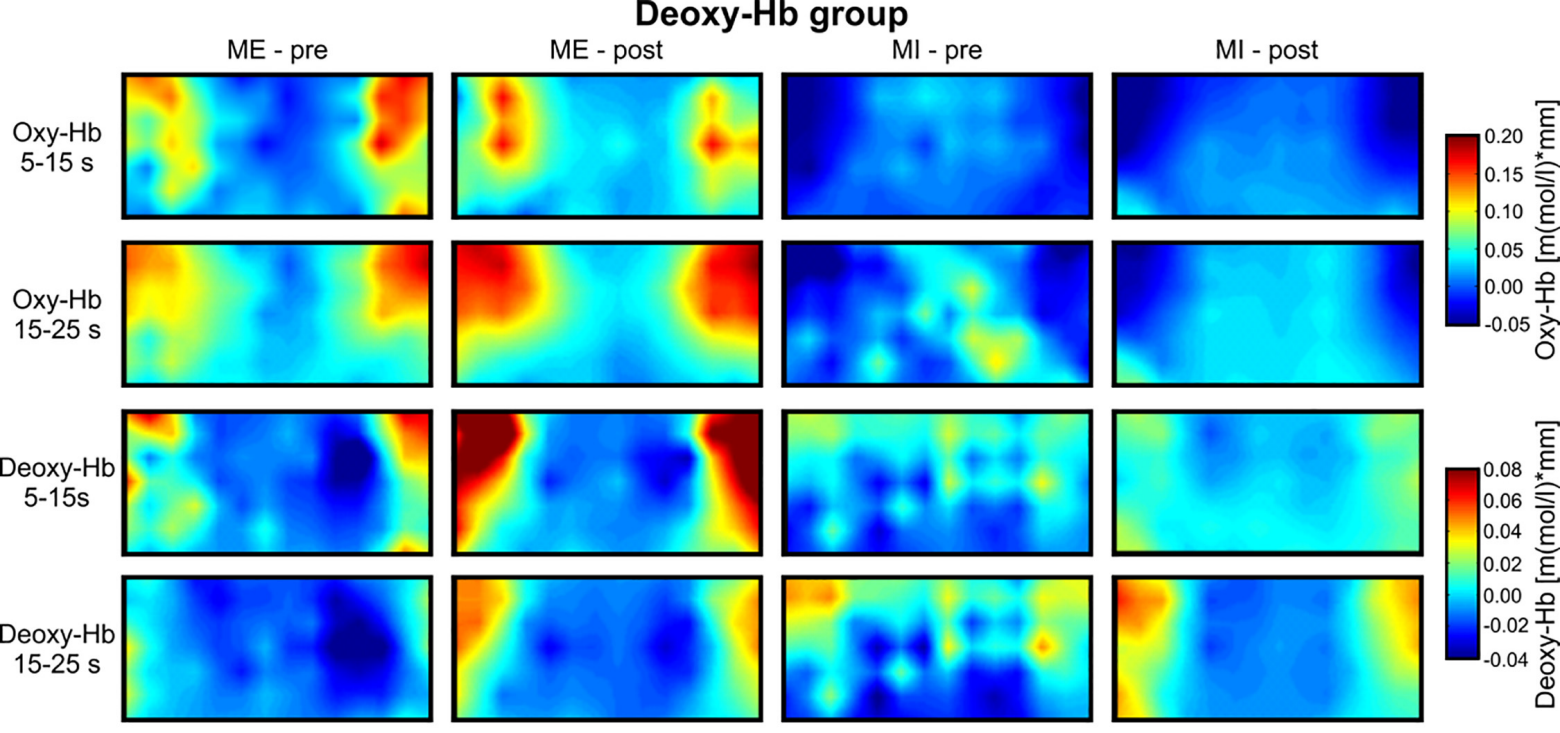
Topographical maps of deoxy-Hb. Grand average topographical maps of oxy- and deoxy-Hb during motor execution (ME) and motor imagery (MI) for the deoxy-Hb group, presented separately for the task interval (second 5–15 after task onset) and the pause interval (second 15–25 after task onset) and for the pre- and post-test. In the upper left map, the 48 NIRS channel locations are additionally marked.

To investigate topographical differences in the NIRS signal change during ME and MI, we used the FDR method [68]. This analysis revealed that during ME and MI, relative concentration changes in oxy- and deoxy-Hb were strongest over channels 1, 4, 27, and 31 (*p* < FDR 0.10) in both groups (see Figs 4 and 5). For changes in deoxy-Hb during ME, channel number 5, 11, and 38 were significant, too. Based on the results of the FDR analysis, channels that showed the strongest signal change during all conditions were merged to two regions of interest (ROI) for further analysis: left inferior frontal region (LF), average channel 1 and 4; right inferior frontal region (RF), average channel 27 and 31. All other channels did not reach the significance level clearly (*p* > FDR 0.10).

The topographical analysis revealed that changes in oxy- and deoxy-Hb during both tasks, MI and ME, were most pronounced over the bilateral inferior frontal gyrus. In further analysis, only NIRS signal changes in LF and RF regions were analyzed. Figs 4 and 5 illustrate the topographical distribution of relative concentration changes in oxy- and deoxy-Hb for the different tasks during the pre- and post-test, for the deoxy-Hb group (Fig 5) and oxy-Hb group (Fig 4).

To analyze differences in brain activation patterns during ME and MI of swallowing over the inferior frontal gyrus between the pre- and post-assessment, ANOVAs were applied. For the deoxy-Hb group, the main-effect PRE-POST was significant for the dependent variable deoxy-Hb (*F*(1,9) = 10.23, *p* < 0.05, *η* ^*2*^ = 0.53). The deoxy-Hb level was more pronounced over the inferior frontal gyrus after the NF training (*M* = 0.05; *SE* = 0.01) when compared to the pre-test (*M* = 0.02; *SE* = 0.01) in the deoxy-Hb group (see Fig 5). The main effect TIME was significant, too (*F*(1,9) = 6.42, *p* < 0.05, *η* ^*2*^ = 0.42). Deoxy-Hb was higher during the task period (*M* = 0.04; *SE* = 0.01) than during the subsequent resting period (*M* = 0.02; *SE* = 0.01). The significant interaction effect TIME*TASK (*F*(1,9) = 10.43, *p* < 0.05, *η* ^*2*^ = 0.54) indicated that changes in the relative concentration of deoxy-Hb were stronger during ME than during MI during the task interval (ME task: *M* = 0.07; *SE* = 0.02; MI task: *M* = 0.01; *SE* = 0.01), but not during the subsequent resting period (ME pause: *M* = 0.02; *SE* = 0.01; MI pause: *M* = 0.03; *SE* = 0.01). The ANOVA for the dependent variable oxy-Hb for the deoxy-Hb group revealed only a significant main effect TASK (*F*(1,9) = 15.08, *p* < 0.01, *η* ^*2*^ = 0.63), showing higher values in oxy-Hb during ME (*M* = 0.13; *SE* = 0.03) than during MI (*M* = -0.06; *SE* = 0.03).

For the oxy-Hb group, the ANOVA for the dependent variable oxy-Hb revealed a significant main-effect of TASK (*F*(1,9) = 55.39, *p* < 0.01, *η* ^*2*^ = 0.86), indicating higher values in oxy-Hb during ME (*M* = 0.22; *SE* = 0.02) than during MI (*M* = -0.01; *SE* = 0.02). All other effects were not significant. For the dependent variable deoxy-Hb, the ANOVA model revealed a significant main effect TASK (*F*(1,9) = 6.46, *p* < 0.05, *η* ^*2*^ = 0.42) and a significant main effect TIME (*F*(1,9) = 48.89, *p* < 0.01, *η* ^*2*^ = 0.846). Since the interaction effect TIME*TASK was significant (*F*(1,9) = 31.41, *p* < 0.01, *η* ^*2*^ = 0.78), too, the interpretation of the significant main effects is redundant. Post-tests revealed that relative concentration changes in deoxy-Hb were comparable between ME and MI during the task interval (ME task: *M* = 0.08; *SE* = 0.01; MI task: *M* = 0.05; *SE* = 0.01), but not during the subsequent resting period (ME pause: *M* = -0.02; *SE* = 0.01; MI pause: *M* = 0.06; *SE* = 0.01).

**Fig 6:**
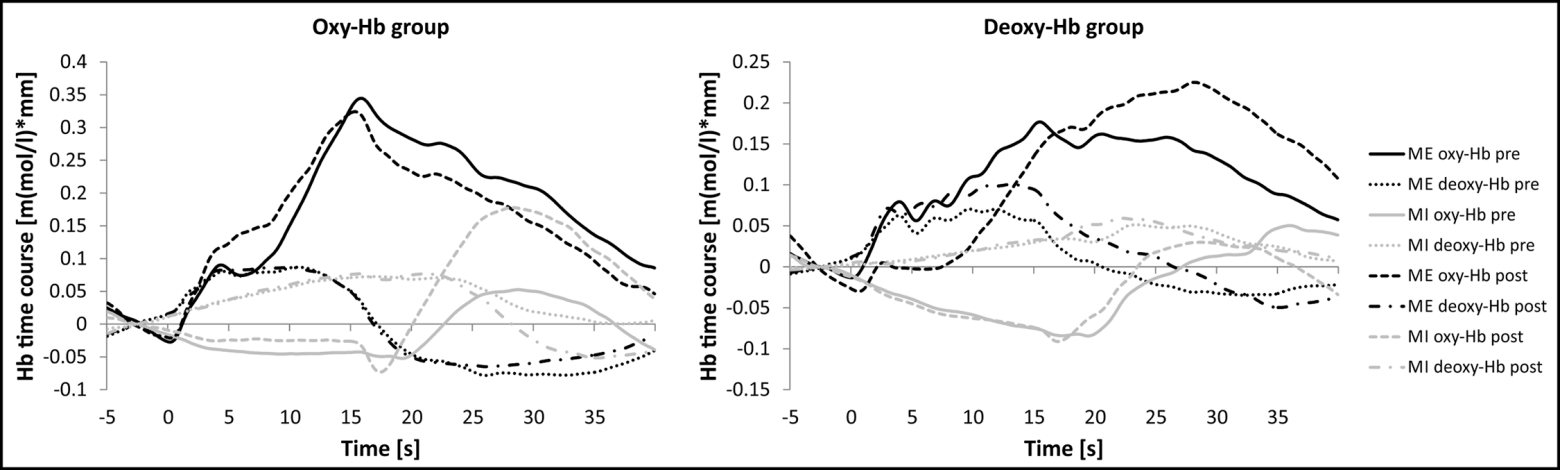
NIRS time course during MI and ME of swallowing. Mean activation changes in oxy- and deoxy-Hb in response to motor execution (ME, black lines) and motor imagery (MI, gray lines) over the inferior frontal gyrus, presented separately for the pre- (full lines) and post-measurement (dotted lines) and the oxy-Hb group (left panel) and deoxy-Hb group (right panel).

In Fig 6, the time course of oxy- and deoxy-Hb during MI and ME is illustrated separately for the pre- and post-assessment and for both NF training groups.

## Discussion

In the present proof-of-principle study, we investigated for the first time the feasibility of NIRS-based NF training using motor imagery of swallowing as mental strategy and its effects on cortical underpinnings of swallowing. Healthy young participants were trained to voluntarily increase either oxy- or deoxy-Hb over swallowing related motor areas during mental imagery of swallowing movements. Participants who were trained to modulate deoxy-Hb successfully increased their deoxy-Hb level over the inferior frontal gyrus during NF training. This voluntary modulation of deoxy-Hb levels led to a more pronounced cortical activation during ME and MI of swallowing during the post- compared to the pre-assessment. In contrast, participants that tried to increase oxy-Hb voluntarily were not able to modulate oxy-Hb over swallowing related brain areas during MI of swallowing. This group also showed no cortical changes when comparing the pre- and post-assessment. In the following, these results and their practical implications are discussed in more detail.

### 4.1 NIRS-based neurofeedback training performance

Results of the present study demonstrate that voluntary control over relative concentration changes in deoxy-Hb during MI of swallowing movements is possible, whereas the concentration level of oxy-Hb could not be increased linearly within seven sessions of NIRS-based NF training. Hence, the deoxy-Hb group was successful in increasing deoxy-Hb over the inferior frontal gyrus by means of NF training. In contrast, the oxy-Hb group was not able to control oxy-Hb. Both groups did not differ in their overall ability to imagine movements as assessed with the MIQ-R questionnaire.

Prior NIRS-based real-time feedback studies only used oxy-Hb as feedback signal while participants imagined hand movements, since oxy-Hb generally exhibits larger signal changes than deoxy-Hb and oxy-Hb is associated with the CBF [27,28]. These studies showed that healthy participants as well as patients with brain lesions can learn enhanced voluntary control over the oxy-Hb signal [1–4]. In the present proof-of-principle study, we used both hemodynamic parameters as feedback signal, since motor imagery of swallowing has never been used as mental strategy in NF training studies before.

When comparing the time course of the NIRS signal change between mental imagination of hand and swallowing movements, there are fundamental differences, which might explain the differences in trainability of oxy- and deoxy-Hb between these two mental imagery tasks. Comparable to the results of our prior NIRS studies, the duration and peak levels of the NIRS signal changes in response to ME and MI of swallowing were longer and later during pre- and post-assessment than for other motor tasks such as ME and MI of hand movements [9,12,23]. Oxy-Hb levels were most pronounced at the end or after the 15 seconds ME and MI task interval. This implies that for motor execution and imagery of swallowing the time course of associated neuronal activity is prolonged compared to other motor tasks [12]. This result is in line with prior NIRS and fMRI studies investigating the hemodynamic response during motor execution of swallowing that found a similar prolonged time course, too [31,37,52,53]. For instance, Hamdy et al. (1999) reported that the BOLD signal had a peak occurring 19+/−3 s after swallowing onset. They tried to explain this with the fact that the whole swallowing process from the mouth to the stomach can take up to 8–12 s. Secondary motor activity in the esophagus may be caused by this long-lasting swallowing event and afferent or efferent feedback loops from cortex to the periphery may lead to such a prolonged cerebral response [31,37]. However, based on the present findings we cannot draw any definitive conclusion why the NIRS time course is prolonged during swallowing tasks at this stage of research.

During MI of swallowing, oxy-Hb levels even decreased and increased again in the subsequent pause interval. Hence, relative concentration changes in oxy-Hb during ME and MI of swallowing showed an opposite trend. During ME of swallowing, oxy-Hb increased, whereas oxy-Hb decreased during MI of swallowing. In contrast, prior NIRS studies found a relative increase of oxy-Hb during MI of hand movements, comparable to the oxy-Hb increase during ME of hand movements [1,4,9,12]. The decrease in oxy-Hb over swallowing related brain areas during MI of swallowing generally reflects a reduction of activation in the corresponding brain area and might be a sign of movement inhibition [12,25,69]. Other NIRS studies investigating cortical correlates of MI and ME of hand movements did not report such differences between relative concentration changes in oxy-Hb during MI and ME [1,3,4,9]. Probably, it is easier to inhibit hand movements than swallowing movements, since the swallowing process contains also involuntary reflexes [29]. Hence, a stronger cortical inhibition indicated by a significant decrease in oxy-Hb in the inferior frontal gyrus might be necessary to avoid active swallowing during MI. In contrast to the relative concentration changes in oxy-Hb, the time course of deoxy-Hb was largely comparable between ME and MI of swallowing during pre- and post-assessment, which is in line with previous findings [12,23]. Both tasks led to an increase in deoxy-Hb over the inferior frontal gyrus bilaterally. We hypothesize that participants of the deoxy-Hb group were more successful in gaining voluntary control over changes in deoxy-Hb than the oxy-Hb group when trying to modulate oxy-Hb, because of these fundamental differences in the time course of these two signals. Inhibiting active swallowing movements during MI of swallowing generally leads to decreased oxy-Hb levels in corresponding motor areas [12,69]. This might have hindered the voluntary increase of oxy-Hb during NF training. Supporting the assumption of motor inhibition processes during MI of swallowing, the deoxy-Hb group showed a significant linear decrease in oxy-Hb levels over the seven NF training sessions. The fact that relative concentration changes in deoxy-Hb behave in a comparable way during ME and MI of swallowing might be a reason for successful NF learning in the deoxy-Hb group. The trainability of deoxy-Hb has been proven successful in prior real-time fMRI studies as well [7,54–56]. There is a large body of evidence showing that participants can learn to voluntarily modulate the BOLD response, which corresponds to the deoxy-Hb signal. These results implicate that deoxy-Hb is more suitable as feedback signal than oxy-Hb for NIRS-based NF studies using motor imagery of swallowing movements as mental strategy.

### 4.2 Effects of NIRS-based NF training on cortical activation patterns during ME and MI of swallowing

The deoxy-Hb group was able to voluntarily modulate the hemodynamic response during NF training and consequently showed a more pronounced activation over the inferior frontal gyrus during the post- compared to the pre-test. The level of deoxy-Hb was increased during both tasks, ME and MI of swallowing, after the NF training compared to the pre-test. Hence, the NIRS-based NF training had an effect on cortical activation patterns responsible for the swallowing process, which might be a sign for neuronal reorganization or neuronal plasticity processes in this group. This result is in line with prior NIRS-based NF studies using mental imagery of hand movements that showed that NF training enhances motor imagery-related cortical activation [1,3,4]. In contrast, the oxy-Hb group was not able to voluntarily modulate the oxy-Hb signal during NF training and consequently showed no changes in brain activation patterns during MI and ME of swallowing when comparing the pre- and post-test.

Supporting the results of our prior study [12], MI and ME of swallowing led to the strongest NIRS signal change over the inferior frontal gyrus bilaterally as assessed during the pre- and post-test. Moreover, the topographical distribution of the NIRS signal change was comparable between the MI and ME task. This area around BA 44 and BA 45, which corresponds to Broca’s area and its homologue in the right hemisphere, is generally considered to subserve motor speech production. However, different studies investigating cortical correlates of swallowing found a strong activation in Broca’s area during swallowing and tongue movement tasks [31,36,37,40]. Sensation of the human mouth and pharynx were localized to BA 44, too [70]. Hence, Broca’s area is also involved in the control of non-speech orofacial sensorimotor behaviors [37,71]. Activation in deeper brain structures, which lie proximal to the inferior frontal gyrus, such as the insular cortex (BA 13), might have caused activation changes over the inferior frontal gyrus during MI and ME of swallowing, too [12]. Various studies consistently report an involvement of the insular cortex in the swallowing process [31,34,36,37,71–73]. In humans, damage to the insula and inner side of the frontal operculum (BA 44, 45) often leads to dysphagia. These results lend support to the role of these brain regions in the swallowing process [41,74,75]. Furthermore, Kern et al. (2001) linked increased cerebral activation patterns around the frontal operculum and insular cortex during the voluntary phase of swallowing to top-down control mechanism such as planning or decision making [30]. Hence, an increased activation in these brain regions during MI of swallowing, as found in the present investigation, might be related to such top-down control processes, which are generally involved during the voluntary/oral swallowing phase.

As already discussed, we found a comparable time course of changes in deoxy-Hb during MI and ME of swallowing, whereas the time course of oxy-Hb was different between MI and ME of swallowing. During MI of swallowing, oxy-Hb decreased, which might be a sign of motor inhibition processes [12,25,69], while during ME of swallowing, oxy-Hb increased. Furthermore, the NIRS signal change was generally more pronounced during ME than during MI. This is in line with findings of previous ME/MI NIRS studies showing increased brain activation patterns during active movement than during mental imagery [9,12].

## Conclusion and Future Directions

Here we show for the first time that (i) MI of swallowing can be used as mental strategy in NF training paradigms, and (ii) NIRS-based NF training leads to cortical reorganization. In the present proof-of-principle study, NF training was performed with healthy young adults that showed no difficulties in swallowing. The question whether NIRS-based NF leads to cortical reorganization in the swallowing cortex and consequently to an improved swallowing function in people with swallowing dysfunction (dysphagia) remains open. Nevertheless, our results provide first evidence for the potential usefulness of NIRS-based NF training in the treatment of dysphagia.
